# Polymorphism of the PAI-1gene (4G/5G) may be linked with Polycystic Ovary Syndrome and associated pregnancy disorders in South Indian Women

**DOI:** 10.6026/97320630013149

**Published:** 2017-05-31

**Authors:** Maniraja Jesintha Mary, Lakshmanan Saravanan, Munuswamy Deecaraman, Melantharu Vijayalakshmi, Vetrivel Umashankar, Jaigopal Sailaja

**Affiliations:** 1 Dr. MGR Educational and Research Institute, Maduravoyal, Chennai; 2 ARC International Fertility Research Center, Perambur, Chennai; 3 Vision Research Foundation, Kamalnayan Bajaj Institute for Research in Vision and Ophthalmology, Sankara Nethralaya Research Institute, Chennai; 4 Genes N Life Healthcare, Hyderabad

**Keywords:** PCOS, Polycystic Ovary Syndrome, PA1-1, Polymorphism

## Abstract

Polycystic Ovary syndrome (PCOS) is the most common endocrine disorder affecting 5 - 10% of all women of reproductive age group. The present research was carried out to study the impact of Plasminogen Activator Inhibitor (PAI-1) 4G/5G polymorphism (rs1799889) in PCOS, and the risk of developing PCOS in South Indian Population. The study was carried out in 60 subjects of South Indian population (30 PCOS and 30 Non PCOS) recruited from ARC Research and Fertility Centre, Chennai, India. Genotype and Allelic frequencies were compared by Fisher exact test, Hardy Weinberg equilibrium. p<0.05 was considered statistically significant. The Genotype frequency difference between PCOS and non-PCOS was observed as statistically non-significant (p=0.4647, OR=1.3077, 95% CI 0.63-2.68). The allelic frequency distribution in Spontaneous Abortion (SAB) cases in total subjects is not found to be statistically significant (p=0. 29), however the PCOS women carrying mutant homozygous and heterozygous genotype are more prone to recurrent pregnancy loss. Out of 17 Implantation failure cases, 23.52% were found to carry mutant homozygous (4G/4G), and 66.66% carried mutant heterozygous (4G/5G), and 5.88% carried wild type homozygous (5G/5G), the allelic difference was highly significant with 4G (62.5%), and 5G (37.5%). P value is highly significant and recorded at p=0.0164. The positive correlation between PAI-1 4G/5G polymorphism and PCOS risk was not observed in this study, however, the correlation between Recurrent Pregnancy Loss (RPL) and Implantation failures were observed in PCOS cases.

## Background

Polycystic ovary syndrome or PCOS is one of the important causes of female subfertility and the most frequent endocrine disorder in women of reproductive age [1]. The PCOS condition is categorized by irregular ovarian morphology and by clinical symptoms like oligo-amenorrhea, anovulation, obesity, fertility related issues, hirsutism, adverse uterine bleeding. The cause of polycystic ovary syndrome is still unclear due to its multifactorial complexity [2]; however, it has been observed and understood that there are several environmental and genetic factors, such as genetic variations like mutations and polymorphisms, differential regulation of genes, and pathways, may contribute to the pathogenesis of PCOS [3]. The information towards the underlying mechanism of PCOS will help in identifying the candidate genes or biomarkers in the disease condition. It has been indicated that differential expression of genes, genetic variations, and other molecular alterations interplay in PCOS and are the target sites for clinical applications, which could be collected from the PCOSDB, repository of candidates genes [4].

Plasminogen activator inhibitor type 1 (PAI-1) is a protein-encoding gene, plays an important role in extracellular matrix remodeling and fibrinolysis (clot dissolving). Increased expression of PAI-1 is found playing a role in autoimmune disorders, cancers and metabolic syndromes. The important function of PAI-1 is to inhibit plasminogen activators (t-PA and u-PA) from converting plasminogen to plasmin, which is responsible for initiating fibrinolysis. The principle is that if there is too much PAI-1 activity, clots will tend to form around, and if there were too little of the PAI-1 activity, the individual would be at an increased risk of bleeding disorders. Hence the role of PAI-1 is very important, as it might have significant impact infertility, autoimmune diseases, and various types of cancers etc. Infertility and sub-fertility is one of the main reasons for PCOS women. The note that PCOS women had an increased risk for early pregnancy loss (EPL) became prominent by various studies carried out on patients with recurrent pregnancy loss (RPL) and IVF treatment.

The PAI-1 (-675 4G/5G) promoter polymorphism (rs1799889) affects transcriptional activity, and is the main inhibitor of the plasminogen activators associated with blood coagulation mechanism, and PAI-1, 4G/4G & 4G/5G, are associated with increased blood concentrations of PAI-1. Earlier studies revealed that the variation 4G/5G polymorphism of the PAI-1 gene is found to be associated with PCOS risk in different populations like Chinese, Caucasian, and has concluded that high PAI-1 levels seem to be linked with first-trimester miscarriage in PCOS women [5]. The association between the PAI-1 4G/5G and Pregnancy complication is significantly important to study. It was earlier reported that the PAI-1 activity is positively associated with the risk of first trimester miscarriage in PCOS women, due to its increased expression of PAI-1 levels associated with the polymorphism, leading to coagulation or clotting, and significant correlation was found in women with polycystic ovary syndrome (PCOS) between elevated levels of PAI-1, and early pregnancy loss (EPL) [6]. In an earlier study, the pregnancy complications with at least one pregnancy, and found a significant association of the 4G/4G PAI-1 polymorphism with prematurity, intrauterine growth restriction (IUGR) [7]. In a subsequent study, the author reconfirmed the presence of the 4G/4G genotype as a risk factor for IUGR and extended their findings to include associations with severe preeclampsia, placental abruption, and stillbirth. They also reported that “the hypofibrinolytic 4G/4G mutation of the PAI-1 gene is frequently associated with the thrombophilic factor V Leiden mutation” which would further increase the risk of problems, related to clotting [8]. The aim of the research is to study and determine the impact of clinical and genetic (PAI-1-675 4G/5G polymorphism (rs1799889)) factors on polycystic ovary syndrome (PCOS) of South Indian Population, and to help the scientific and medical community towards treating the PCOS affected women in a more appropriate and best possible way [9].

## Methodology

### Study group

The study was conducted at ARC Research and Fertility Hospital, Perambur, Chennai, India. Sixty (60) women, including both PCOS and Non-PCOS (mean age 24.77 ± 3.03 years) were included in the study. Non-PCOS and PCOS are grouped based on Rotterdam criteria 2003. The Ethics Committee of ARC Fertility Research Centre, approved the study after fulfilling the selection criteria, informed written consent was obtained from the subjects.

### Genetic analysis - Genotyping of PAI-1 4G/5G polymorphism

Genomic DNA was isolated from peripheral blood leukocytes using the Phenol chloroform method. A DNA Fragment containing part of the 5’ untranslated region UTR) and the full region of exon 1 was amplified with the primers: Forward, 5’ – TGTCTGTGTCTGGAGGAAGAGGAT-3’ and reverse, 5’ - CCCAACAGAGGACTCTTGGTCTTT-3’ as described previously (Table 1). Since (-675) 4G/5G polymorphism is a polymorphism based on the insertion-deletion of a G allele in PAI-1 promoter, the product of the PCR was 98 bp for the 4G allele and 99 bp for the 5G allele. Polymorphism chain reaction (PCR) was run in a total volume of 10 μl containing 1 μl of genomic DNA, 0.25 μl of each primer, 5 μl of Master mix and 3.5 μl of water. The PCR protocol was conducted as follows: Initial Denaturation at 94°C for 10 sec, followed by 40 cycles consisting of 10 sec of denaturation at 98°C, 30 sec of annealing at 63°C, and 60 sec of extension at 72°C; and a final single extension of 10 min at 72°C. For 5′-UTR, the genotyping was carried out by direct sequencing on an ABI 3130 automated sequencer (Applied Biosystems). Genotyping for the PAI-1 4G/5G polymorphism was performed by the PCR-restriction fragment length polymorphism (RFLP) assay. Digestion was visualized following electrophoresis on a 3% agarose gel containing ethidium bromide. DNA samples were selected for direct sequencing to validate the variation.

### Statistical analysis

Data was analyzed using computer program Excel 2010 for all mathematical and statistical analysis. The difference between results were considered statistically significant if P<0.05. The mean value and the standard deviation were calculated for the Biochemical data. Fisher's exact test was used when appropriate to detect the association between genotypic variants in the PAI-1 Gene (4G/5G). The PCOS risks were determined by the odds ratio (OR) and its corresponding 95% confidence intervals (CIs). The results of serum hormone levels, age and BMI are reported as the mean ± standard deviation. Genotype distribution analysis between PCOS and Non PCOS was carried out by Fisher's exact test. All the statistical analysis was performed using the statistical program Excel, MedCalc (www.medcalc.org), VASSAR (www.vassarstats.net), Hardy Weinberg equilibrium. P<0.05 was considered to indicate a statistically significant difference. All values are expressed as mean + standard error of the mean.

## Results & Discussion

**Figure 1:**
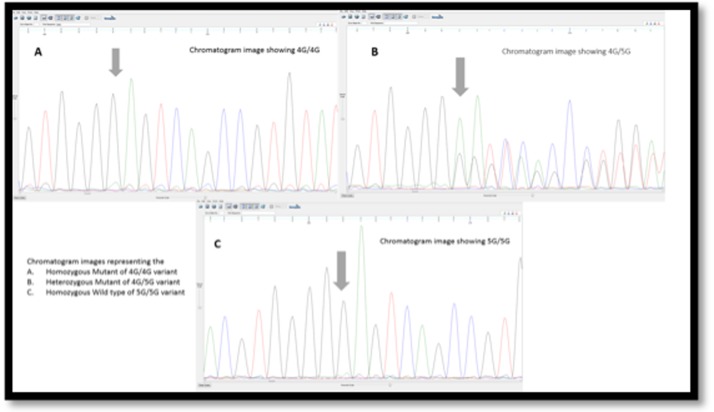
Chromatogram results of mutants is shown

Polycystic ovary syndrome is a highly intricate and multifactorial disorder thought to be the impact of a complex interaction between immunological, genetic and environmental factors. As the PCOS found to be a heritable disorder within the families, it highlights the importance of genetic factors in the pathogenesis of the PCOS risk and development [9]. Several case–control studies have been carried out to determine PAI-1 4G/5G polymorphism and PCOS in other population, including Caucasian, Chinese, Turkish etc., however there is no research done on PAI-1 gene polymorphisms and risk of PCOS in South Indian women. This study adds to the current knowledge of genetic susceptibility by investigating for the first time an interactive association between PAI-1 4G/5G and on the incidence of PCOS in South Indian women. The results underline the importance of this genetic polymorphism and its contribution towards the risk of PCOS and associated fertility and pregnancy disorders. In the present study, 4G/5G polymorphism in the 3'-UTR region of the PAI-1 gene and the risk of developing PCOS in South Indian women was investigated. According to the results observed by electrophoresis of PCR and RFLP product, the genotype of every individual for 4G/5G polymorphism of PAI-1 gene were determined. The chromatogram results of 4G/4G homozygous mutant (Figure 1A), 4G/5G heterozygous mutant (Figure 1B), and 5G/5G wild type (Figure 1C) are represented.

### Genotype and Allele frequency distribution

The results of the genotype and allele frequency distribution, odds ratio, confidence interval, p value for each polymorphism are summarized in the tables (Table 2, Table 3). The distribution of different allelic forms and their corresponding values of significance are enumerated. It is evident that there is no significant difference between the distribution of the genotypes between PCOS and Non PCOS women. The PAI-1 (4G/5G) Genotype and Allelic frequencies for PCOS and Non PCOS group, the Dominant model was studied (Table 4). Genotype frequencies between PCOS and Non PCOS subjects were compared by Fisher exact test. p<0.05 was considered statistically significant. Out of 30 PCOS cases, 6 (20%) are found to carry Mutant Homozygous (4G/4G), 18 (60%) are found to carry Mutant heterozygous (4G/5G), and 6 (20%) are wild type homozygous (5G/5G). Out of 30 non-PCOS cases, 3 (10%) subjects are with mutant homozygous, 20 (67%) with mutant heterozygous, and 7 (23%) are with wild type homozygous. Although the mutant form is slightly elevated in PCOS, statistically significant difference was not observed between PCOS and non- PCOS (p=0.65).

Allele frequencies between PCOS and Non PCOS subjects were compared by Hardy Weinberg equilibrium. p<0.05 was considered statistically significant. The difference between PCOS and non- PCOS was not statistically significant (p=0.4647, OR=1.3077, 95%CI 0.63-2.68). Genotype and Allele frequencies between PCOS and Non PCOS subjects (Dominant model) were compared by Fisher exact test and Hardy Weinberg equilibrium. The difference between PCOS and non-PCOS was statistically non-significant (p=0.7542, OR=1.2174, 95%CI 0.3554-4.1705).

PAI-1 (4G/5G) Genotype and Allelic frequencies in Recurrent Pregnancy Loss (more than one pregnancy loss) was evaluated in total subjects (including PCOS and Non PCOS subjects) were compared by Fisher exact test and Hardy Weinberg equilibrium. Out of 9 SAB cases 1 (11.11%) found to carry mutant homozygous (4G/4G), and 6 (66.66%) carried mutant heterozygous (4G/5G), and 2 carried wild type homozygous (5G/5G). The allelic frequency distribution is not found to be statistically significant (p=0. 29). Similarly PAI-1 (4G/5G) Genotype and Allelic frequencies in implantation failure, and recurrent implantation failure was evaluated in total subjects had one or more IVF failure (including PCOS and non PCOS subjects). Genotype and Allele frequencies between PCOS and Non PCOS subjects were compared by Fisher exact test and Hardy Weinberg equilibrium. Out of 17 Implantation failure cases 4 (23.52%) were found to have mutant homozygous (4G/4G), and 12 (66.66%) with mutant heterozygous (4G/5G), and 1 (5.88%) with wild type homozygous (5G/5G), the allelic difference was highly significant with 4G (62.5%), and 5G (37.5%). P value is highly significant and recorded at p=0.0164

Glueck investigated the complications in several women with at least one pregnancy, and found a significant association of the 4G/4G PAI-1 polymorphism with intrauterine growth restriction (IUGR) and prematurity [7], although our results do not support the earlier results of, it is still very interesting to observe the 4G/4G and 4G/5G are more in the cases and controls with early pregnancy loss. Considering these results, we interpret that 4G/4G polymorphism in the PAI-1 gene could be a thrombophilic variant leading to abortion, and analysis of this mutation and other susceptibility factors are recommended in patients with recurrent pregnancy loss (Table 5) and with recurrent implantation failure (Table 6). It was observed that the 4G allele frequency is moderately higher in women with PCOS than in controls and 4G/4G genotype frequency followed a similar pattern.

In earlier studies 4G allele was identified as one of the risk factors for the development of various diseases such as myocardial infarction, autoimmune disorders in Indian, Chinese and Caucasian populations respectively. Previous studies presented the PAI-1 activity were positively associated with the risk of first trimester miscarriage in PCOS women [8]. The previous pregnancy and associated miscarriages, including missed abortion, Spontaneous abortion, and recurrent pregnancy loss were elevated in PCOS women with 4G/4G and 4G/5G mutant condition. PCOS women carrying 4G/5G mutation are prone to pregnancy risk at an early stage due to increased level of PAI-1 leading to clotting disorders [10].

In the present study, the frequency of the 4G allele in both the group together, with recurrent pregnancy loss (RPL) was 45%, and the frequency of 5G allele was, 55%, which did not show a significant difference (p=0.29). Similarly the frequency of distribution of 4G allele was studied in the total group who had at least 1 implantation failure through IVF treatment. The results showed a significant difference between 4G and 5G allelic distributions of 62.5% and 37.5%, respectively, with the p value 0.0164 (Table 4). Considering these results, 4G/4G polymorphism in the PAI-1 gene might be a thrombophilic variant leading to abortion, and further analysis of this mutation and other susceptibility factors are recommended in patients with RPL. The results from the study reveals the PAI-1 4G/5G may be associated with pregnancy disorders. Although the difference is not highly significant between PCOS cases and Non PCOS controls, it is still important to observe the impact of mutation.

## Conclusion and Future scope

In conclusion, the present study reports for the first time an association between PAI-1 4G/5G polymorphism and risk of implantation failures and moderate risk of early and recurrent miscarriages in PCOS women of South Indian population. Although the 4G/5G are not found to be associated with PCOS risk (p value 0.65), however, it’s been noted that PCOS women carrying 4G/5G mutation are prone to early pregnancy loss, including Missed Abortion (MA), Recurrent Pregnancy Loss (RPL) and Spontaneous Abortion (SAB) and Implantation Failure (IF). It is essential to carry this study with bigger sample to get more insight. Early detection of the PAI-1 4G/5G might help in early diagnosis and focused treatment. This point towards the importance of necessary treatment even before the conception starts. It might help to treat these patients much in advance with blood thinners like heparin, aspirin (the suggested drugs) in order have more successful pregnancies. This study brings an insight, and also gives a way to expand this research to a larger population to obtain more promising results for the women facing fertility problems. The results produced by this study need to be supplemented by further studies and research conducted on larger patient groups and using more advanced techniques that should be available in the future.

## Conflict of Interest

There is no conflict of interest in this research

## Figures and Tables

**Table 1:** PAI-1 Gene (4G/5G) Primers

	Gene	Primer sequence 5׳-3׳
Forward	PAI-1	5׳-TGTCTGTGTCTGGAGGAAGAGGAT-3׳
Reverse	PAI-1	5׳-CCCAACAGAGGACTCTTGGTCTTT-3׳

**Table 2:** PAI-1 (4G/5G) genotype frequencies for PCOS and Non PCOS group

Genotype	PCOS		Non PCOS		P-value
	n (30)	%	n (30)	%	
4G/4G	6	20	3	10	0.65
4G/5G	18	60	20	67	
5G/5G	6	20	7	23	
Total	30	100	30	100	
Genotype frequencies between PCOS and Non PCOS subjects were compared by Fisher exact test. p<0.05 was considered statistically significant.					

**Table 3:** PAI-1 (4G/5G) allelic frequencies for PCOS and Non PCOS group

Allele	PCOS (n=30) %	Non PCOS (n = 30) %	P-value	Odds Ratio	95% CI
4G	(30) 50%	(26) 43%	0.4646	1.3077	0.6372 - 2.6837
5G	(30) 50%	(34) 57%			
Total	1	0			
Allele frequencies between PCOS and Non PCOS subjects were compared by Hardy Weinberg equilibrium. p<0.05 was considered statistically significant.					

**Table 4:** PAI-1 (4G/5G) Genotype and Allelic frequencies for PCOS and Non PCOS group (Dominant model)

Genotype	PCOS		Non PCOS		P-value	Odds Ratio	95% CI
	n (30)	%	n (30)	%			
4G/4G + 4G/5G	24	80	23	77	0.7542	1.2174	0.3554, 4.1705
5G/5G	6	20	7	23			
Total	30	100	30	100			
Genotype and Allele frequencies between PCOS and Non PCOS subjects (Dominant model) were compared by Fisher exact test and Hardy Weinberg equilibrium. p<0.05 was considered statistically significant.							

**Table 5:** PAI-1 (4G/5G) Genotype and Allelic frequencies Recurrent Pregnancy Loss

Total Pregnancy Loss Cases within the total subjects including PCOS and Non PCOS					
	SAB (n=9)	%	Allele frequency		P value
4G/4G	1	11.11%	4G	8 (44.44%)	0.29
4G/5G	6	66.66%	5G	10 (55.56%)	
5G/5G	2	22.22%			
Genotype and Allele frequencies between PCOS and Non PCOS subjects were compared by Fisher exact test and Hardy Weinberg equilibrium. p<0.05 was considered statistically significant.					

**Table 6:** PAI-1 (4G/5G) Genotype and Allelic frequencies in Implantation Failure

Total Implantation Failure Cases within the total subjects including PCOS and Non PCOS					
	IMP (n=17)	%	Allele frequency		P value
4G/4G	4	23.52%	4G	20 (62.5%)	0.0164
4G/5G	12	66.66%	5G	14 (37.5%)	
5G/5G	1	5.88%			
Genotype and Allele frequencies between PCOS and Non PCOS subjects were compared by Fisher exact test and Hardy Weinberg equilibrium. p<0.05 was considered statistically significant.
